# Differences in the aggressiveness of familial versus sporadic non-medullary thyroid cancer: An unresolved controversy

**DOI:** 10.20945/2359-4292-2026-0018

**Published:** 2026-02-16

**Authors:** Fabíola Yukiko Miasaki, Teresa Cristina Santos Cavalcanti, Hans Graf, Edna Teruko Kimura, Peter Andreas Kopp, Gisah Amaral de Carvalho

**Affiliations:** 1 Divisão de Endocrinologia (Serviço de Endocrinologia e Metabologia do Paraná), Departamento de Clínica Médica, Universidade Federal do Paraná, Curitiba, PR, Brasil; 2 Departamento de Patologia, Universidade Federal do Paraná, Curitiba, PR, Brasil; 3 Departamento de Biologia Celular e do Desenvolvimento, Instituto de Ciências Biomédicas, Universidade de São Paulo, São Paulo, SP, Brasil; 4 Division of Endocrinology, Diabetology and Metabolism, Centre Hospitalier Universitaire Vaudois, University of Lausanne, Lausanne, Switzerland

**Keywords:** Thyroid neoplasms, thyroid cancer, papillary, genetic predisposition to disease, neoplastic syndromes, hereditary

## Abstract

**Objective:**

Familial non-medullary thyroid cancer (FNMTC) is defined as non-medullary thyroid cancer occurring in two or more first-degree relatives, without features of known hereditary syndromes. Although familial predisposition is well established, its clinical behavior remains debated. This study aimed at characterizing familial cases compared to sporadic non-medullary thyroid cancer (SNMTC).

**Subjects and methods:**

FNMTC and SNMTC patients were recruited from the Endocrine Division (SEMPR) of the Federal University of Paraná, Brazil, and private endocrine clinics in Curitiba, Paraná, Brazil (2000-2019). Baseline, histopathological, and clinical data were analyzed using SPSS Statistics 26.0. Statistical comparisons employed chi-square, Student’s t test, and Mann-Whitney U test, as appropriate. Post hoc power analysis was performed using G*Power 3.1.9.7, and R 2025.05.0.

**Results:**

We analyzed 39 FNMTC and 119 SNMTC patients. Papillary thyroid carcinoma was the predominant histological type in both groups. FNMTC patients were diagnosed at a younger age (38.5 ± 14.2 vs. 46.6 ± 13.8 years, p = 0.003) and more frequently presented with lymph node metastases at diagnosis (46.2% vs. 21.8%, p = 0.007), with a 4.57-fold increased risk. Despite these differences, long-term outcomes did not differ significantly between groups. An earlier disease onset in subsequent generation suggests a possible anticipation phenomenon.

**Conclusion:**

These findings suggest that FNMTC patients may present with earlier onset and higher rates of lymph node involvement, underscoring the need for thorough preoperative lateral neck evaluation. In view of a possible anticipation phenomenon, cervical ultrasound screening might be considered starting in adolescence.

## INTRODUCTION

The occurrence of thyroid cancer in twins was first reported in 1953 by Firminger and Skelton ^(1)^. Soon afterwards, in 1955, Robinson and Orr also reported 24-year-old monozygotic twins affected by isolated papillary thyroid cancer ^(2)^. However, the concept of Familial Non-Medullary Thyroid Carcinoma (FNMTC) has only been firmly established in the last decades ^(3)^. FNMTC is defined by two or more first-degree relatives affected by non-medullary thyroid carcinoma (papillary, follicular, oncocytic, anaplastic) without syndromic signs (Cowden syndrome, Gardner syndrome/Familial Adenomatous Polyposis, Carney complex, Werner syndrome) ^(4,5)^.

Patients with FNMTC account for 1.5%-10% of thyroid cancer cases ^(6-12)^, and many controversies still surround this entity. Multicentricity and lymph node metastases were observed more commonly in FNMTC by some ^(10,11,13,14)^ but not all authors ^(7,8,15)^. Similarly, some but not all have reported a higher incidence of recurrences, as well as a compromised disease-specific survival in FNMTC compared to sporadic non-medullary thyroid cancer (SNMTC) ^(16)^.

Syndromic forms of FNMTC have well-documented genetic etiologies. Mutations in *PTEN*, *APC*, *WRN*, and *PRKAR1A* are associated with Cowden syndrome, Gardner syndrome/Familial Adenomatous Polyposis, Werner syndrome, and Carney complex, respectively. In contrast, non-syndromic FNMTC has no single well-defined genetic predisposition, although an autosomal dominant inheritance pattern with incomplete penetrance is likely ^(17)^. Although multiple loci and variants have been described in FNMTC, most of these alterations appear to be restricted to a limited number of families or are private to specific kindreds. FNMTC has been linked to several loci, including *TCO*, *NMTC1*, *PRN1*, *4q32*, *6q22*, *8p23.1-22*, and *8q24*, as well as variants in *NKX2-1*, *FOXE-1/PTCSC2*, *SRGAP1*, *SRRM2*, *NOP53*, *HAPB2,* and *PDPR*. Additionally, genes associated with the shelterin complex, such as *TINF2*, *POT1,* and *ACD* are thought to be implicated in its pathogenesis ^(18,19)^. Moreover, multigenic mechanisms have also been postulated as a mechanism to confer PTC predisposition ^(20)^. However, the extent of their contribution to FNMTC susceptibility remains mostly uncertain and requires additional investigation.

Given the ongoing controversies concerning the clinical behavior of FNTMC, the absence of high penetrance mutations outside of the syndromic forms, and the observed heterogeneity in FNMTC families in terms of age-dependent penetrance and expressivity, it remains of interest and importance to thoroughly characterize such families. This may ultimately provide novel insights into mechanisms of carcinogenesis, impact clinical management, and permit to establish structured recommendations for screening, and, in some cases, refine treatment strategies.

With the goal of contributing to a better understanding of FNMTC as a clinical entity, this study aimed at characterizing the clinical presentation at diagnosis and treatment response of familial cases, in comparison to sporadic thyroid cancer.

## SUBJECTS AND METHODS

Patients with FNMTC and SNMTC diagnosed between 2000 and 2019 were included in this study. FNMTC was defined as the presence of non-medullary thyroid cancer (papillary, follicular, oncocytic, or anaplastic) in two or more first-degree relatives. Furthermore, patients with signs indicative or suggestive of syndromes associated with thyroid cancer such as Cowden syndrome, Gardner syndrome/Familial Adenomatous Polyposis, Carney complex, Werner syndrome, or ataxia-telangiectasia were excluded. A specific questionnaire and clinical examination were performed to exclude syndromes associated with thyroid carcinomas in the Endocrine Division (SEMPR), Department of Internal Medicine, Federal University of Parana. SNMTC was defined as the absence of a history of thyroid cancer in the family and cases were selected from our institutional database.

FNMTC patients were identified through the SEMPR’s dedicated database and were also recruited from private endocrine clinics in Curitiba between 2015 and 2019. The thyroid cancer database of SEMPR has been established in 2007 and has formal approval by the Hospital’s Ethics Committee (CAAE: 48391715.8.0000.0096). In 2007, all information was collected retrospectively and, since then, prospectively. Some affected relatives of the index cases of these FNMTC kindreds have received treatment outside of Curitiba (Rio de Janeiro (Brazil), Londrina (Brazil), Bogota (Colombia)). In these instances, the medical records were obtained from the treating physicians and/or clinical institutions for chart review.

The control cohort comprising SNMTC is formed by patients followed at the Federal University of Parana, Curitiba, a tertiary hospital, and consists of patients that have been diagnosed and followed exclusively at this institution. Patients referred because of advanced metastatic disease were excluded from the control cohort to avoid potential bias. No children or adolescents under the age of 14 were included in this study.

Data about the type of surgery, patient characteristics (sex, age at diagnosis), histological characteristics (type, size, presence/absence of capsule, capsular invasion, angio-lymphatic invasion, perineural invasion, gross extrathyroidal extension, presence of lymph node involvement, extra nodal extension, and distant metastases) were collected. These data were compiled, and each case was classified according to the American Joint Committee on Cancer (AJCC) TNM classification, 8^th^ edition ^(21)^, and the 2009 American Thyroid Association (ATA) risk stratification system ^(22)^, rather than the version from 2015 ^(23)^, because detailed data on lymph node size and/or the number of lymphovascular invasions was missing.

An expert thyroid pathologist revised all cases with unclear pathology reports and excluded one case of non-invasive follicular neoplasm with papillary-like features (NIFTP). Response to therapy was determined with parameters including biochemical markers (thyroglobulin (Tg) and antithyroglobulin antibodies), as well as imaging studies (ultrasound, scintigraphy, computerized tomography), and classified according to the 2015 ATA guidelines (excellent response, biochemical incomplete response, structural incomplete response, and indeterminate response) ^(23)^.

To study the possibility of an anticipation phenomenon – an earlier onset of disease manifestations in subsequent generations – we only included FNMTC kindreds with documented DTC in a parent and some of their offspring.

The statistical analyses were performed using SPSS Statistics for Windows (Version 26.0, IBM Corporation, Armonk, New York). For qualitative variables, we used the Chi-square/Fisher’s test; and for quantitative analyses, the T-student test or the Mann-Whitney U test as indicated. Post hoc power analyses were performed using G*Power 3.1.9.7 and R 2025.05.0.

This project was approved by the Ethics Committee of the Clinical Hospital at the Federal University of Parana (CAAE: 40511015.5.0000.0096).

## RESULTS

In total, we recruited 42 familial cases, of which three were excluded. Because the focus of this study was on FNMTC without any tumors in other organs, one kindred with two cases was excluded due to concurrent breast cancer and heterozygosity for a pathogenic *ATM* variant (*ATM* c.3848T>C; p.L1283P) ^(24)^. Another family was excluded due to a strong familial history of lung cancer.

**Figure 1.**
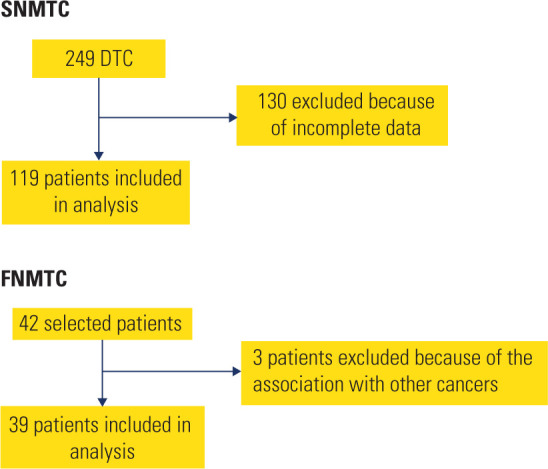
Flow diagram of patient exclusion criteria for SNMTC and FNMTC groups.

SNMTC: sporadic non-medullary thyroid cancer; FNMTC: familial non-medullary thyroid cancer.

For SNMTC cases, we evaluated 249 patients for enrollment. However, 130 had insufficient data leaving a final SNMTC cohort of 119 subjects (**Figure 1**).

### Baseline characteristics

There were no sex differences between the FNMTC and SNMTC groups, with the majority being female (79.5% *vs.* 88.2%, *p* = 0.187). Management strategies were also similar: all FNMTC patients (100%) and most SNMTC patients (96.6%) underwent total thyroidectomy. Central neck dissection was only performed in patients with clinical evidence of lymph node metastases but not in a prophylactic manner.

FNMTC patients were diagnosed at a younger age than SNMTC patients (38.5 ± 14.2 *vs.* 46.6 ± 13.8, *p* = 0.003). Furthermore, when analyzing the age of diagnosis between the first and second generations of FNMTC, a significant difference was found, with diagnosis at a younger age in the second generation (49.7 ± 11.6 *vs.* 32.5 ± 13.1 years, *p* = 0.002). The third generation was not included in this analysis, as it comprised only a single patient.

The mean available follow-up period was shorter for FNMTC patients compared to SNMTC subjects (7.0 ± 3.6 *vs.* 10.4 ± 5.3 years, *p* < 0.001). In addition, all SNMTC patients were treated within the Public Health System. However, as patients with oncologic conditions receive treatment priority, this did not appear to affect the timing of surgery or subsequent treatments.

A summary of patient characteristics is provided in **Table 1**.

**Table 1.** Baseline characteristics of patients with SNMTC versus FNMTC

	SNMTC	FNMTC	p-value
Age (years old)	46.65 ± 13.77	38.74 ± 14.48	0.003 (power 93.8%)
Sex (female)	105 (88.2%)	31 (79.5%)	0.187 (power 24.8%)
Follow-up (years)	10.40 ± 5.32	7.05 ± 3.58	<0.001
Public Health System patients	119 (100%)	9 (23.1%)	<0.001 (power 100%)
Total thyroidectomy	115 (96.6%)	39 (100%)	0.573 (power 0.1%)

Student’s t-test for independent samples (age), Fisher’s exact test for categorical variables; p < 0.05.

SNMTC: sporadic non-medullary thyroid cancer; FNMTC: familial non-medullary thyroid cancer.

### Histopathological characteristics

In both groups, papillary thyroid cancer was the predominant histological type: (104/119, 87.4% in SNMTC *vs.* 38/39, 97.4% in FNMTC, *p* = 0.178). Interestingly, the mean tumor size was smaller in the FNMTC group (18.28 ± 13.56 mm *vs.* 25.27 ± 20.39 mm, *p* = 0.084), although not statistically significant. For other histopathological characteristics (capsular invasion, angiolymphatic invasion, extrathyroidal extension, multicentricity), there were no statistically significant differences between the two groups.

### Lymph node and distant metastatic disease

Despite similar primary tumor characteristics, FNMTC patients had a significantly higher rate of lymph node metastasis at initial surgery compared to SNMTC patients (46.2% *versus* 21.8%, *p* = 0.007). Univariate and multivariate analyses were performed to assess for potential confounding factors. Univariate analysis showed that the main factors for lymph node involvement were family history, age at diagnosis, angiolymphatic invasion and minimal extrathyroidal extension. Multivariate analysis revealed that FNMTC patients had a 4.57-fold higher risk of lymph node metastasis (**Table 2**). Additionally, younger age was associated with a higher risk of lymph node involvement (*p* = < 0.001; OR = 0.94). Minimal extrathyroidal extension was also correlated with an increased risk of lymph node metastasis.

**Table 2.** Multivariate analysis for risk of lymph node metastasis

Predictor^a^	Classification	Total	N		P^b^	OR (IC95%)^b^
			No Mean ± dp or n (%)	Yes Mean ± dp or n (%)		
Age	Mean ± dp	158	47.2 ± 13.3	38.2 ± 14.9	<0.001	0.94 (0.91-0.98)
Familiarity	SNMTC		93 (78.2%)	26 (21.8%)		
	FNMTC		21 (53.8%)	18 (46.2%)	0.003	4.57 (1.67-12.5)
Angiolymphatic invasion	0 (ref)	70	55 (78.6%)	15 (21.4%)		
	1	68	43 (63.2%)	25 (36.8%)	0.091	2.23 (0.88-5.68)
Extrathyroidal invasion	0 (ref)	123	99 (80.5%)	24 (19.5%)		
	1	27	11 (40.7%)	16 (59.3%)	<0.001	10.15 (3.33-30.93)

a Predictors were selected according to the statistical significance in the univariate analysis. Perineural invasion was excluded due to the small number of cases exhibiting this feature (only 7).

b Multivariate Logistic Regression Model and Wald Test, p < 0.05.

The prevalence of distant metastatic disease at initial diagnosis did not differ between the SNMTC and FNMTC groups (9.2% *versus* 0%, *p* = 0.067).

The TNM stages of both groups (FNMTC and SNMTC) is shown in **Table 3** (American Joint Committee on Cancer Staging System 8^th^ edition) ^(21)^.

**Table 3.** Characteristics of SNMTC versus FNMTC

		SNMTC	FNMTC	p-value
**Histological characteristics**				
Size (mm)		25.27 ± 20.39 (1.6-95)	18.28 ± 13.56 (5-60)	0.084 (power: 68.4%)
Histology	Papillary	104 (87.4%)	38 (97.4%)	0.178
	Follicular	9 (7.6%)	1 (2.6%)	(power: 36.1%)
	Oncocytic	6 (5%)	-	
Multicentric disease		39/112 (34.8%)	18/39 (46.2%)	0.251 (power: 20.2%)
No capsular invasion		21/108 (19.4%)	6/37 (16.2%)	0.365 (power: 22.4%)
Angiolymphatic invasion		50/102 (49%)	18/36 (50%)	1.000 (power: 3.6%)
Minimal extrathyroidal extension		22/113 (19.5%)	5/37 (13.5%)	0.471 (power: 8.2%)
Gross extrathyroidal extension		7/113 (6.2%)	0/39 (0%)	0.191 (power: 4.5%)
Initial clinical presentation				
Cervical lymph node involvement		26 (21.8%)	18 (46.2%)	0.007 (power: 79.3%)
Distant metastases		11 (9.2%)	0 (0%)	0.067 (power: 41.6%)
AJCC/TNM according to 8th edition				
I		101 (84.9%)	39 (100%)	
II		13 (10.9%)	0 (0%)	
IVa		1 (0.8%)	0 (0%)	
IVb		4 (3.4%)	0 (0%)	

SNMTC: sporadic non-medullary thyroid cancer; FNMTC: familial non-medullary thyroid cancer; AJCC/TNM: American Joint Committee on Cancer/Tumor, Node, Metastasis system.

### Radioiodine therapy

The frequency of radioiodine therapy was comparable between the FNMTC and SNMTC groups (*p* = 0.799). The administered activities were lower on average in the FNMTC group (3.49 ± 3.06 GBq *vs.* 5.5 ± 6.03), although the difference did not reach statistical significance (*p* = 0.054). The administered activities ranged from zero to 38.48 GBq in the SNMTC group, and from zero to 14.8 GBq in FMNTC patients.

### Response to therapy and recurrences

There were no significant differences in response to therapy between the two groups (*p* = 0.230), nor in the incidence of recurrences (*p* = 0.187).

## DISCUSSION

Although it has been known for decades that thyroid cancer can be highly heritable ^(25,26)^, leading to the concept of FNMTC, its clinical behavior and outcomes compared to SNMTC remain a subject of debate. While some studies suggest that FNMTC exhibits a more aggressive presentation, others report no significant differences in disease manifestation and progression ^(10,15,27,28)^.

The findings presented here suggest that FNMTC is associated with a higher prevalence of lymph node metastasis at initial diagnosis (46.2% *vs.* 21.8%, *p* = 0.007). Similar results have been reported in several studies ^(9,10,12)^; although others did not make this observation ^(8,11,13,15,29,30)^. One might argue that the surgeons involved were more inclined to perform central lymph node dissection, potentially influencing the results, as lymph node micrometastases are highly prevalent in PTC. Yet, as outlined above and consistent with current guidelines ^(23,31)^, prophylactic central lymph node dissection was not performed in this cohort. Nonetheless, the involvement of multiple surgeons makes it impossible to rule out performance bias.

Although the follow-up period is relatively short, we observed a higher recurrence rate in the FNMTC group compared to SNMTC patients (20.5% *vs*. 11.8%). However, this difference did not reach statistical significance (*p* = 0.187). This finding is consistent with previous reports, which suggested that while FNMTC may exhibit more lymph node metastasis. But the long-term oncologic outcomes do not differ significantly from those of SNMTC patients ^(7,12)^.

In the cohort presented here, the mean age at diagnosis was significantly lower in the FNMTC group than in the SNMTC group (38.5 ± 14.2 *vs.* 46.6 ± 13.8, *p* = 0.003). Previous studies have consistently reported younger age at diagnosis in FNMTC patients ^(3,6,10,13,15,27,29)^, though this trend was not observed in families with only two affected members ^(32)^. One potential confounder in our analysis might be ascertainment bias. Many of the FNMTC patients had better access to healthcare, facilitating earlier detection and diagnosis of thyroid abnormalities. Furthermore, the diagnosis of thyroid cancer in one individual may prompt screening of family members, leading to earlier detection, even for small carcinomas.

The possibility of anticipation, in which disease onset occurs earlier in successive generations, has been suggested for FNMTC ^(30)^. To explore this, we analyzed the age difference between generations within FNMTC families and observed a significant trend toward earlier diagnosis in subsequent generations (*p* = 0.002), with some cases diagnosed more than a decade earlier. Although intriguing, this finding should be interpreted with caution and requires further confirmation. As discussed, early case identification and screening in families with known thyroid cancer may contribute to detection and ascertainment bias. This, in turn, increases the risk of a Type I error in assessing age-of-onset anticipation, and no statistical analysis can eliminate this potential limitation ^(33)^.

Several factors may explain the discrepancies in the literature regarding initial presentation and clinical course of FNMTC. One major challenge is the classification of familial cases. Charkes postulated that using a criterion of only two affected individuals per family poses a high risk (62%-69%) of misclassifying sporadic cases as familial due to the high incidence of DTC ^(34)^. However, other studies have suggested that even families with only two affected members may exhibit more aggressive disease ^(32)^. The inclusion of both two-member and larger families in most studies, including ours, further complicates comparisons. In our cohort, 56% of FNMTC cases originated from families with at least three affected members. Comparing patients from families with at least three affected members to those from families with only two affected individuals, we found no significant differences in sex, age, or histological characteristics (data not shown).

FNMTC studies encompass families from diverse genetic backgrounds. In the absence of a known high penetrance gene, it is likely that FNMTC exhibits genetic heterogeneity, with multiple low-penetrance genetic variants at multiple loci contributing to disease susceptibility ^(18,32)^. Environmental factors may also play a role in disease manifestation and progression, further complicating efforts to delineate the underlying mechanisms of FNTMC.

Regarding treatment outcomes, no significant differences were observed between the SNMTC and FNMTC groups in terms of the ATA response-to-therapy reclassification. This suggests that the therapeutic interventions were equally effective in both groups. However, we acknowledge the limitations of our study, including a relatively short follow-up period (mean: 7.1 ± 3.6 years) and a limited sample size. Nevertheless, our findings align with previous studies reporting comparable treatment responses between FNMTC and SNMTC patients ^(7,12)^.

In conclusion, according to the data obtained from this cohort, FNMTC appears to be associated with a higher prevalence of lymph node metastases compared to SNMTC. Therefore, a thorough preoperative evaluation of the lateral neck remains essential.

Furthermore, our findings suggest that there may be an anticipation phenomenon in FNMTC, although detection and ascertainment bias remain a possible alternative explanation. Yet, based on these findings, structured ultrasound evaluation beginning at approximately 15 years of age may be recommended for individuals in subsequent generations.

## Data Availability

datasets related to this article will be available upon request to the corresponding author.
